# Spatial clustering of high load ocular *Chlamydia trachomatis* infection in trachoma: a cross-sectional population-based study

**DOI:** 10.1093/femspd/ftx050

**Published:** 2017-05-03

**Authors:** Anna Last, Sarah Burr, Neal Alexander, Emma Harding-Esch, Chrissy H. Roberts, Meno Nabicassa, Eunice Teixeira da Silva Cassama, David Mabey, Martin Holland, Robin Bailey

**Affiliations:** 1 Clinical Research Department, London School of Hygiene and Tropical Medicine, Keppel Street, London WC1E 7HT, UK; 2 Disease Control and Elimination Theme, Medical Research Council Unit The Gambia, PO Box 273 Banjul, Atlantic Boulevard, Fajara, The Gambia; 3 MRC Tropical Epidemiology Group, London School of Hygiene and Tropical Medicine, Keppel Street, London WC1E 7HT, UK; 4 Programa Nacional de Saúde de Visão, Ministério de Saúde Publica, PO Box 50, Avenida de Unidade Africana, Bisssau, Guiné Bissau

**Keywords:** *Chlamydia trachomatis*, bacterial load, spatial clustering, trachoma, Guinea Bissau

## Abstract

*Chlamydia trachomatis (Ct)* is the most common cause of bacterial sexually transmitted infection and infectious cause of blindness (trachoma) worldwide. Understanding the spatial distribution of *Ct* infection may enable us to identify populations at risk and improve our understanding of *Ct* transmission. In this study, we sought to investigate the spatial distribution of *Ct* infection and the clinical features associated with high *Ct* load in trachoma-endemic communities on the Bijagós Archipelago (Guinea Bissau). We collected 1507 conjunctival samples and corresponding detailed clinical data during a cross-sectional population-based geospatially representative trachoma survey. We used droplet digital PCR to estimate *Ct* load on conjunctival swabs. Geostatistical tools were used to investigate clustering of ocular *Ct* infections. Spatial clusters (independent of age and gender) of individuals with high *Ct* loads were identified using local indicators of spatial association. We did not detect clustering of individuals with low load infections. These data suggest that infections with high bacterial load may be important in *Ct* transmission. These geospatial tools may be useful in the study of ocular *Ct* transmission dynamics and as part of trachoma surveillance post-treatment, to identify clusters of infection and thresholds of *Ct* load that may be important foci of re-emergent infection in communities.

## Teaser

We identified clusters of high load Ct infections suggesting that high load infections may be important in transmission, which may have implications in trachoma elimination strategies and surveillance.

## INTRODUCTION

*Chlamydia trachomatis* is the leading infectious cause of blindness and the most common sexually transmitted bacterium. Trachoma is caused by infection with ocular strains of *C. trachomatis* and manifests as distinct clinical syndromes, beginning with an acute self-limiting keratoconjunctivitis which may progress to chronic inflammatory disease with subsequent conjunctival scarring and blinding sequelae. A spectrum of disease severity exists, which can be graded objectively (Dawson, Jones and Tarizzo 1981; Thylefors *et al*. 1987).

The World Health Organization (WHO) advocates the implementation of the SAFE strategy (Surgery for trichiasis, Antibiotics for active infection, Facial cleanliness to prevent disease transmission and Environmental improvement to increase access to water and sanitation) for trachoma elimination. Mass Drug Administration (MDA) of azithromycin aims to clear infection from communities such that trachoma ceases to be a public health concern (Kuper *et al*. 2003).

Understanding the spatial distribution of disease and infection in trachoma-endemic regions is increasingly recognised in national trachoma control programme planning, enabling the identification of at-risk populations and prioritisation of target areas for control and implementation and optimal scaling of SAFE (Baker *et al*. 2010; Clements *et al*. 2010; Smith *et al*. 2011, 2013a,b). It may also be important in understanding transmission, transmission thresholds and the dynamics of spread and recovery from infection following intervention.

Spatial analysis provides powerful methods to study the relationship between active trachoma and ocular infection with *C. trachomatis*. The spatial relationship between disease and infection defined over space is termed spatial dependence. This is measured as the existence of statistical association (dependence) between disease and infection associated with location. To explore spatial dependence, measures of spatial autocorrelation are used. Spatial autocorrelation assumes that near objects are more related than distant objects. In the context of infectious diseases, this concept may be applied to explore the relationship between infection and disease where the spatial distribution can be used to define underlying processes such as exposure or transmission. Spatial regression models can thus be used to understand the epidemiology of active trachoma and *C. trachomatis* infection.

Global spatial autocorrelation statistics are used to describe the overall spatial patterns in the data. Local indicators of spatial association take actual values of observations in the context of adjacent values allowing mapping and definition of clusters at exact locations. This allows for the examination of small-scale heterogeneity and identification of statistically significant clusters and outliers of similar observations in space. In the context of global spatial patterns described above, this provides us with a greater understanding of the relationship between infection and disease.

In this study, we applied molecular and geostatistical tools to investigate the spatial epidemiology of *C. trachomatis* infection and active trachoma in a population-based study conducted in a trachoma-hyperendemic treatment-naïve population from the Bijagós Archipelago of Guinea Bissau in West Africa. This is the first study to use local indicators of spatial association using individual-level *C. trachomatis* load data in the context of spatial dependence to investigate the relationship between infection and disease. This is important in understanding the epidemiology of ocular *C. trachomatis* infection and may inform further studies on *C. trachomatis* transmission dynamics, which are fundamental to successful trachoma elimination and surveillance strategies.

## MATERIALS AND METHODS

### Ethical approval

This study was conducted in accordance with the declaration of Helsinki. Ethical approval was obtained from the Comitê Nacional de Ética e Saúde (Guinea Bissau), the LSHTM Ethics Committee (UK) and The Gambia Government/MRC Joint Ethics Committee (The Gambia). Written informed consent was obtained from all study participants or their guardians on their behalf if they were children. A signature or thumbprint is considered an appropriate record of consent in this setting by the above ethical bodies. After survey completion, all communities on the study islands were treated with a single height-based dose of oral azithromycin in accordance with WHO and national protocols.

### Study area

**Figure 1.**
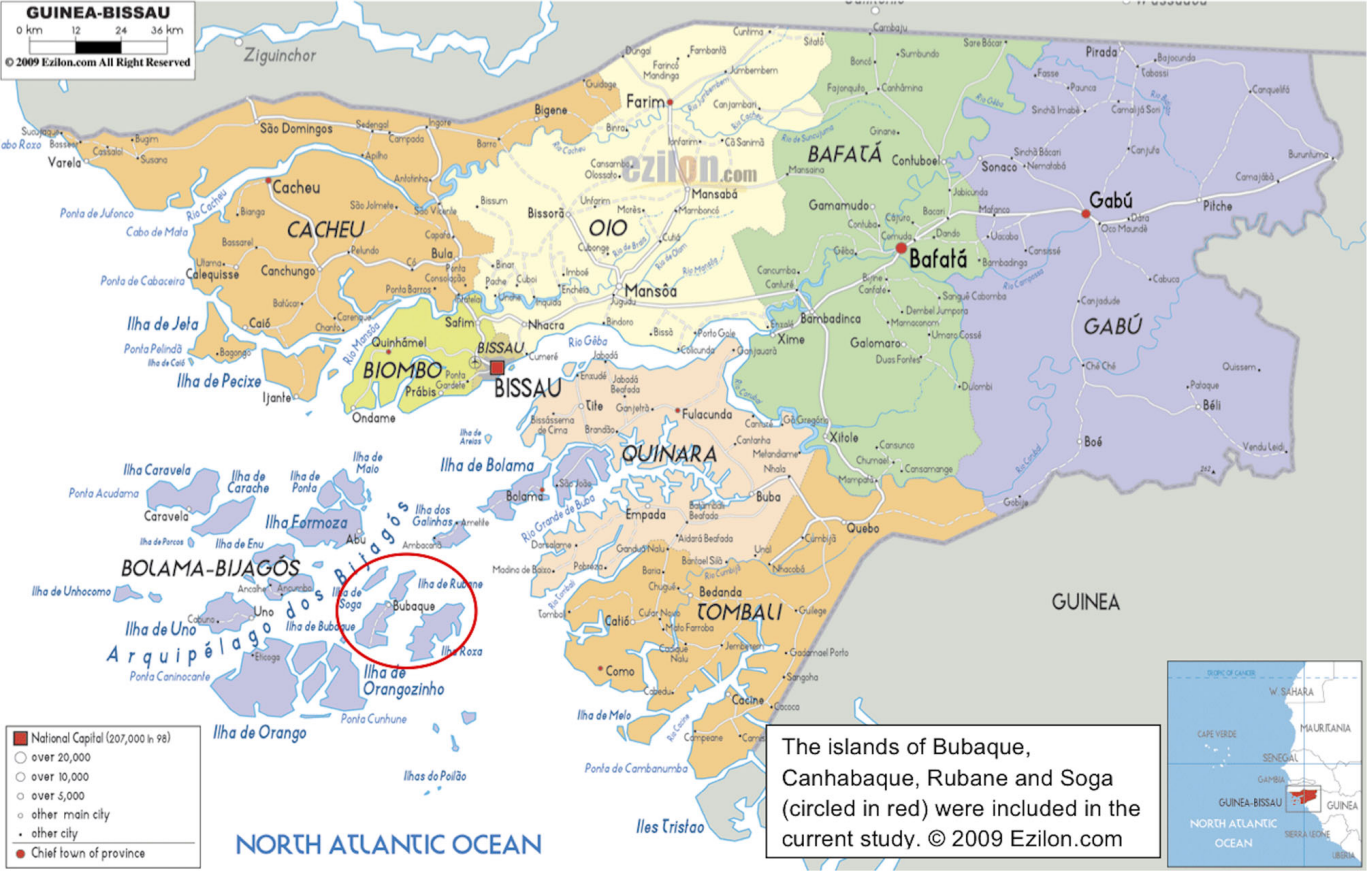
The Bijagós Archipelago, Guinea Bissau (© Ezilon 2009). The islands of Bubaque, Canhabaque, Rubane and Soga (circled in red) were included in the current study.

The Bijagós Archipelago has a total area of more than 10 000 km^2^ and lies between N 11^0^38΄19.68″ and N 10^0^51΄40.32″, W 16^0^29΄38.40″ and W 15^0^27΄17.28″. The surface area covers ∼900 km^2^, of which 350 km^2^ is mangrove forest (Van der Linde and Dankin 1998). Maximum altitude is 50 m. The climate is humid and tropical, with a rainy season from May to November, when average monthly rainfall is 400 mm^3^ (World Bank 2013). Mean monthly temperature is 27.3°C (25.1°C–29.2°C), with peak temperatures prior to the rainy season. There are 88 islands and islets of which ∼20 are permanently inhabited. The remainder are inhabited periodically for seasonal agriculture and traditional initiation ceremonies. The study was conducted on four islands of the archipelago (Fig. 1). These four islands comprise a total rural population of 5613 (National Population Census, 2010, Instituto Nacional de Estatística, Guiné-Bissau) and a total area of 215 km^2^.

### Study design and study population

Trachoma survey methodology and this study population have been described previously (Edwards *et al*. 2008; Harding-Esch *et al*. 2010; Roberts *et al*. 2013; Last *et al*. 2014a,b). To satisfy adequate geospatial representation at village level, we included all 38 villages on the four study islands and randomly sampled one in five households (with a minimum of five per village) from each. All were sampled if there were fewer than six households in the village. Small villages are thus over-represented by the minimum sampling criteria imposed. Data were geocoded at household and village level using the Garmin eTrex H handheld Global Positioning Systems (GPS) unit (Garmin Ltd, UK).

### Clinical examination and conjunctival sampling

A single validated examiner assessed each participant using the WHO simplified and modified FPC grading systems (Dawson, Jones and Tarizzo 1981; Thylefors *et al*. 1987). In the modified FPC system, follicles (F), papillae (P) and conjunctival scarring (C) are each assigned a separate grade from 0 to 3. FPC grades of F2/3 or P3 equate to a diagnosis of active trachoma (TF (follicular trachoma) or TI (inflammatory trachoma) by the WHO simplified system) and a grade of C2/3 (and in some cases C1) equates to a diagnosis of TS (trachomatous scarring). A trachoma grade was assigned to the upper tarsal conjunctivae of each consenting participant using adequate light and a ×2.5 binocular magnifying loupe. Both methods were used in order that study data should be comparable to data used by trachoma control programmes and research studies requiring detailed information related to disease severity and have been used previously in similar settings (Burton *et al*. 2003, 2006).

Samples were taken from the left upper tarsal conjunctiva of each participant with Dacron swabs (Fisher Scientific, UK) using a well-tolerated standardised procedure described in previous studies (Keenan *et al*. 2010; Stare *et al*. 2011; Roberts *et al*. 2013; Last *et al*. 2014a,b).

### Detection and quantitation of *Chlamydia trachomatis* ocular infection

DNA extraction and droplet digital PCR (ddPCR) (Bio-Rad Laboratories, Hemel Hempstead, UK) were conducted as described previously (Roberts *et al*. 2013; Last *et al*. 2014a,b). We used *C. trachomatis* plasmid-based ddPCR to diagnose infection and a single-copy pathogen chromosomal gene (*omcB*) to estimate pathogen load in each plasmid-positive sample (Roberts *et al*. 2013; Last et al. 2014). The plasmid-based screening PCR included primers for human DNA (RPP30). A sample was deemed adequate if sufficient quantities of RPP30 DNA were present on PCR as defined previously (Roberts *et al*. 2013; Last *et al.* 2014).

Estimated quantities of *omcB* (*C. trachomatis* load) and plasmid are expressed as copies/swab. This is a method that we have previously employed for similar analyses (Solomon *et al*. 2003; Burton *et al*. 2003; Faal *et al*. 2006; Andreasen *et al*. 2008; Last et al. 2014; West *et al*. 2014). Data reported by Solomon *et al*. (2004) (showing a clear reduction in *C. trachomatis* load and disease severity in a trachoma-endemic community in Tanzania following mass drug treatment with azithromycin (MDA)), and Alexander *et al*. (2005) (showing a reduction in community *C. trachomatis* load (calculated using the same method) following MDA), provide evidence that measuring *C. trachomatis* load as copies/swab in this context is appropriate. We do not report *C. trachomatis* load per eukaryotic cell since inflammatory cells are attracted to the conjunctiva in the presence of ocular *C. trachomatis* infection which would be included amongst sampled cells and may artefactually decrease the *C. trachomatis* load. This is of particular concern in active trachoma, where inflammation can be intense and result in high loads of human cellular material on a swab. This phenomenon has also been noted in urogenital *C. trachomatis* infection (Jalal *et al*. 2011).

### Statistical analysis

*Chlamydia trachomatis* quantitation data were processed as described previously (Roberts *et al*. 2013; Last et al. 2014). GPS data were downloaded into MapSource v16.16.3 (Garmin Ltd). All data were double entered into a customised database (Microsoft Access 2007) and discrepancies resolved through reference to source documents. Data were cleaned and analysed in STATA 13 (Stata Corporation, College Station, TX, USA). Statistical significance was determined at the 5% level.

#### Mixed effects linear regression models of *Chlamydia trachomatis* load and clinical phenotype

*Chlamydia trachomatis* load data were log(e) transformed where indicated. The geometric mean of load, respective standard error (SE) and 95% confidence intervals (CI) were calculated. An analysis of variance (ANOVA) with pairwise comparisons was used to compare load across detailed clinical phenotypes. Assessment of group differences and multiple comparisons were adjusted for using the Scheffé correction (Ruxton and Beauchamp 2008). Associations between load and detailed clinical phenotype were examined using univariable and multivariable mixed effects linear and logistic regression models accounting for household-level clustering detected in previous studies (Last *et al*. 2014).

### Geostatistical analyses

Geocoded data were projected into UTM Zone 28N. ArcGIS 10.1 (ESRI Inc., USA) and the R statistical package v3.0.2 (The R Foundation for Statistical Computing, http://www.r-project.org using spdep, automap and nlme packages) were used for all geostatistical analyses (Bivand 2010; Hiemstra *et al*. 2009; Pinheiro *et al*. 2014). In the following analyses, the zone of indifference is used to define adjacency. This method assumes that each observation has local influence that decreases with distance beyond a critical distance cut-off, resulting in an adapted model of impedance, or distance decay, such that all features have an impact on all other features, but this impact decreases with distance. The crucial cut-off used in this study is derived from the distance over which spatial autocorrelation occurs in these data and relates to the village boundaries, assuming impedance as described above. Previously we have shown that clustering exists at this level in these communities (Last *et al*. 2014), which supports using this threshold. This method is appropriate for point data and takes into account the extent of spatial autocorrelation in selecting data-driven threshold cut-offs (Chang 2010).

#### Spatial autocorrelation

The Moran's I statistic was used to evaluate global spatial autocorrelation in the distribution of the household-level prevalence of disease and infection. The values of Moran's I range between –1 and +1. A value close to –1 indicates negative autocorrelation (complete dispersion), while a value close to +1 indicates positive autocorrelation (clustering) of features. A value close to 0 suggests random arrangement. Z-scores and *P*-values are assigned to ascertain whether spatial autocorrelation is statistically significant (Moran 1950). Since the Moran's I statistic can be sensitive to skewed distributions, a permutation test was used to verify the results. This uses Monte Carlo simulations to generate a Moran's I sampling distribution (Cliff and Ord 1981).

Empirical semivariograms were used to obtain the distance range over which spatial autocorrelation occurs using the average squared distance between paired data values against the distance (or lag) separating the pairs to estimate the spatial covariance structure of the data to inform the geostatistical models (Diggle, Tawn and Moyeed 1998).

Linear and logistic mixed effects regression models were also applied to examine the effect of spatial autocorrelation on clustering. We used the covariance structure to compare models with and without a spatial component. The log likelihood, Akaike information criterion (AIC) and Bayesian information criterion (BIC) were used to compare models (Zucchini 2000).

#### Local indicators of spatial association: cluster–outlier analyses

Clustering and outlier analysis was applied using the local (Anselin) Moran's I statistic in ArcGIS 10.1 Spatial Statistics Toolbox (ESRI Inc., USA). The zone of indifference was used to define adjacency and the Euclidean distance threshold was derived from empirical semivariograms as described above. The Euclidean distance threshold was thus the distance over which the highest positive spatial autocorrelation for *C. trachomatis* infection existed. Together, these account for the geography (islands with populated (villages) and non-populated areas) (Anselin 1995). Individuals’ *C. trachomatis* loads were used as the variable of interest. Since these populations and areas are small with little ecological variation, this single threshold value is sufficient. A positive value for I indicates that a feature has adjacent features with similarly high or low attributable values (a cluster). A negative value for I indicates that adjacent features have dissimilar values and that this feature is an outlier. Cluster–outlier analysis identifies spatial clusters of features with high or low values and spatial outliers by calculating Moran's I, Z-scores, *P*-values and a code to represent the cluster type. Cluster types are defined as clusters of high values (HH, high-high), clusters of low values (defined as above) (LL, low-low), a high value outlier surrounded by predominantly low values (HL, high-low) and a low value outlier surrounded by predominantly high values (LH, low-high). A group of geospatially proximal infections where the bacterial load values are not statistically similar is defined as a non-significant cluster.

In this analysis, ‘low’ values include both low values related to low load infections and null (zero) values related to uninfected individuals. To explore this, these analyses were conducted first on the whole data set, including uninfected individuals with a ‘zero’ value for infectious load, and then on infected individuals only.

## RESULTS

Trachoma and *Chlamydia trachomatis* infection prevalence is hyperendemic in this population and is described elsewhere in detail (Roberts *et al*. 2013; Last et al. 2014). All swab samples in the study were positive for the presence of the human DNA target RPP30. *Chlamydia trachomatis* load was skewed, with the majority of cases having low copy numbers (<1000 copies/swab)*.* Log-(e) transformation removed the skew (skewness –0.106, *P = 0.5454*) in these analyses.

### *Chlamydia trachomatis* bacterial load and clinical disease severity in individuals with *Chlamydia trachomatis* infection

**Figure 2.**
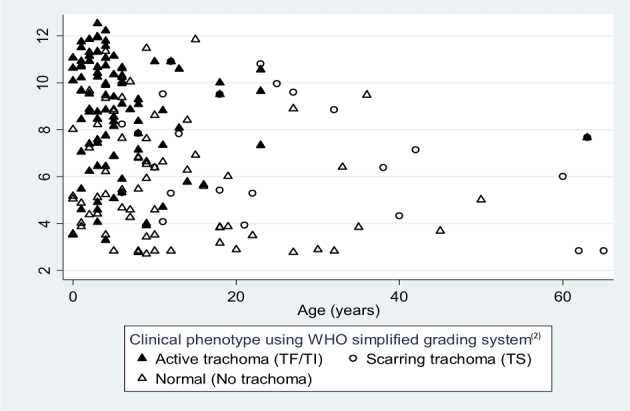
*Chlamydia trachomatis* load by age and clinical phenotype in infected individuals. The y axis shows the natural logarithmic scale of *C. trachomatis* bacterial load (*omcB* copies/swab).

The geometric mean of estimated *omcB* copies/swab present in clinically normal conjunctivae (F0/P0/C0) was 294 copies/swab (95% C.I. 165–524). In clinically active trachoma, it was 8562 copies/swab (95% C.I. 5412–13546). Significantly higher loads were detected in individuals with increasing F and P scores (Table 1). *Chlamydia trachomatis* load by age and clinical phenotype is shown in Fig. 2. The majority of infections with high loads were in children under 10 years of age with active trachoma.

**Table 1.** Estimated *C. trachomatis* load (*omcB* copies/swab) and ANOVA^a^ by detailed clinical phenotype in infected individuals.

Clinical		*omcB* copies/swab	95% CI	*P-Value*					
phenotype	*n* ^b^	(geometric mean)	(SE geometric mean)	(ANOVA)			Median	Min	Max
Normal (F0P0C0)	71	294	165, 524	*Baseline*			176	15	96333
Active trachoma (TF and/or TI)	92	8562	5412, 13546	*P <* ***0.00001***			14236	17	274835
Scarring trachoma (TS)	19	928	280, 3074	*P = 0.4069*			2142	17	49125
Follicular score (F)									
0	91	438	251, 762	*Baseline*			227	15	202632
1	20	1288	448, 3697	*P = 0.324*	*Baseline*		1710	34	96333
2	27	3212	1264, 8165	*P =* ***0.002***	*P = 0.624*	*Baseline*	3203	27	140693
3	46	19870	2832, 25927	*P* < ***0.0001***	*P* < ***0.0001***	*P* = ***0.018***	22767	323	274835
Inflammatory score (P)									
0	46	122	68, 218	*Baseline*			67	15	41059
1	70	1534	871, 2702	*P <* ***0.0001***	*Baseline*		1469	16	202632
2	46	10413	5461, 19857	*P <* ***0.0001***	*P <* ***0.0001***	*Baseline*	18569	17	274835
3	22	14053	5550, 35581	*P <* ***0.0001***	*P <* ***0.0001***	*P = 0.964*	21864	34	158548
Scarring score (C)									
0	155	1902	1207, 2996	*Baseline*			2095	15	274835
1	11	449	111, 1816	*P = 0.438*	*Baseline*		204	34	11556
2	9	990	192, 5111	*P = 0.927*	*P = 0.941*	*Baseline*	589	76	54651
3	9	2475	253, 24192	*P = 0.995*	*P = 0.608*	*P = 0.923*	7023	17	49125

a Scheffé correction used for multiple comparisons.

b *n* = number of individuals with quantifiable *C. trachomatis* bacterial load.

### Spatial structure of active trachoma and *Chlamydia trachomatis* infection

**Figure 3.**
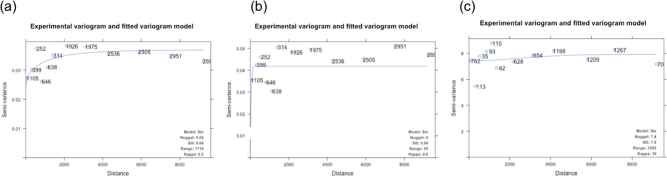
Empirical semivariograms and fitted models for household prevalence of (**a**) ocular *C. trachomatis* infection, (**b**) active trachoma and the distribution of (**c**) ocular *C. trachomatis* bacterial load. (a) Unadjusted household prevalence of *C. trachomatis* infection. (b) Household prevalence of active trachoma in 1–9 year olds. (c) Ocular *C. trachomatis* bacterial load. Prevalence data were log transformed (ln(ln+1)) due to significant negative skew. Active trachoma is defined by TF/TI by the WHO Simplified Grading System (Thylefors *et al*. 1987) (F2/F3 or P3 by the Modified FPC Grading System; Dawson, Jones and Tarizzo 1981). Distance is indicated in metres. Model fit with the smallest residual sum of squares (Matern, M. Stein's Parameterization (Ste)). All values of Kappa (smoothing parameter of the Matern model) tested. Nugget, sill, range and Kappa are all estimated from the data.

Significant positive spatial autocorrelation was evident for *C. trachomatis* infection (Moran's I = 0.19, *P < 0.0001*) but not active trachoma (Moran's I = 0.07, *P = 0.0659*). Semivariograms demonstrate that autocorrelation in infection is negligible in distances greater than 1719 m (Fig. 3). We found no evidence of spatial autocorrelation in the distribution of *C. trachomatis* load (Moran's I = 0.05, *P = 0.4464*).

*Chlamydia trachomatis* load was the strongest predictor of clinically active trachoma. In accordance with the semivariograms, including spatial structure in multivariable mixed effects regression analyses for active trachoma and *C. trachomatis* infection improves the fit of the models (Table 2).

**Table 2.** Multivariable mixed effects logistic regression analysis showing the effect of spatial dependence on clinically active trachoma and ocular *C. trachomatis* infection.

Model	Predictor variables	*n*	AIC*^a^*
*C. trachomatis* infection			
No spatial^b^	Age	1426	854.8
Spatial^c^			**801.8**
No spatial	Age	1426	546.4
Spatial	Active trachoma^d^	163	**495.6**
Active trachoma			
No spatial	Age	1426	697.9
Spatial			**659.0**
No spatial	Age	1426	389.5
Spatial	*C. trachomatis* infection	224	**362.3**
No spatial	Age	1426	251.2
Spatial	*C. trachomatis* load^e^	180	**232.7**

a AIC = Akaike information criterion.

b With household or village as cluster variables.

c Including of spatial structure.

d Defined as TF/TI using the WHO simplified grading system (Thylefors *et al*. 1987).

e Defined as log-(e) *omcB* copies/swab.

Inflammatory and follicle scores were strongly associated with bacterial load. Inclusion of the spatial structure in models predicting *C. trachomatis* load improved the fit, but the effect of spatial dependence becomes undetectable when age and disease severity scores are included, thus resolving residual spatial variation (Table 3).

**Table 3.** Multivariable mixed effects linear regression analysis of factors predictive of *C. trachomatis* load (*omcB* copies/swab) in infected individuals.

Model	Variable	*n*	OR^a^	95% CI	*P-Value*	*AIC* *^b^*	*BIC* *^c^*	*loglik* *^d^*
Null*^e^*								
						884.8	894.3	–439.4
Clustering								
Household*^f^*						884.3	893.8	–439.1
Village*^f^*						882.1	892.0	–438.2
Spatial*^g^*						799.7	834.8	–388.8
Final*^h^*								
Including age and disease severity								
Spatial						802.6	844.1	–388.3
No spatial						**797.7**	**829.6**	**–388.8**
Age	0–5 years	87	2.60	0.99, 6.87	**0.052**			
	6–10 years	45	0.70	0.24, 2.05	0.509			
	11–15 years	15	1.77	0.43, 7.20	0.427			
	>15 years	37	1.00	–	–			
Disease severity	Inflammatory grade (P)							
	0	46	1.00	–	–			
	1	70	7.54	3.12, 18.20	**<0.0001**			
	2	46	22.8	8.15, 63.9	**<0.0001**			
	3	22	30.9	9.39, 101.50	**<0.0001**			
	Follicular grade (F)							
	0	91	1.00	–	–			
	1	20	1.29	0.43, 3.84	0.649			
	2	27	2.20	0.82, 5.88	0.114			
	3	46	7.78	3.16, 19.15	**<0.0001**			

*Chlamydia trachomatis* load is defined as log-(e) *omcB* copies/swab. There was no evidence of heteroscedasticity of residuals (Breusch-Pagan/Cook Weisberg test for heteroscedasticity in the final model (χ^2^ = 0.44, *P = 0.5079*)).

a Exponentiated coefficient.

b AIC = Akaike information criterion.

c BIC = Bayesian information criterion.

d loglik = Log likelihoood.

e Null model with dummy cluster variable.

f Including household or village as cluster variables on outcome.

g Including spatial structure.

h Final model including covariates with and without adjustment for spatial structure.

### Cluster and outlier analysis of *Chlamydia trachomatis* infection

**Figure 4.**
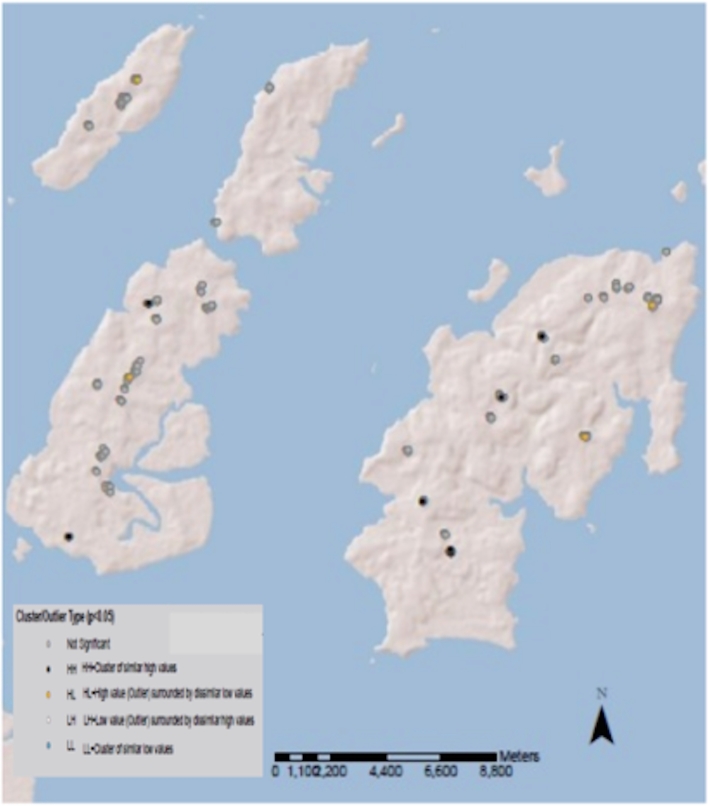
Clusters and outliers of high load ocular *C. trachomatis* infections. *Chlamydia trachomatis* load log transformed (ln(ln+1)) due to significant negative skew. Statistically significant positive values for the Local Moran's I statistic indicate clustering with similarly high (H-H) or low (L-L) values. Negative values indicate that neighbouring observations have dissimilar values and that this observation is an outlier (H-L or L-H). H-H clusters and H-L outliers are observed. There are no L-L clusters. Observation values represent *C. trachomatis* load. Adjacency is defined by the zone of indifference.

High load infections were clustered with other high load infections (HH clusters) (Fig. 4). Outliers where there was a single low load infection amidst predominantly high load infections (HL) were also demonstrated. There was no clustering of low load infections (or zero values from uninfected individuals) (LL clusters), and there were no statistically significant outlying high loads surrounded by predominantly low loads (LH). Analyses were conducted on the whole data set and infections only as described above. There were no individuals with infections below 10 000 *omcB* copies/swab noted within an HH cluster or as an HL outlier.

## DISCUSSION

The majority of ocular *Chlamydia trachomatis* infections occur in children, who have the highest loads and most severe active trachoma. However, infection predominantly occurs at low bacterial loads in the population overall. Similar findings have been observed in other studies (Solomon *et al*. 2003; Burton *et al*. 2006; Faal *et al*. 2006), though we found a greater prevalence of infection across all age groups typical of hyperendemic settings. Almost half the individuals with quantifiable infection had a normal clinical phenotype, though the mean *C. trachomatis* load was significantly lower than in those with active trachoma, which is consistent with other studies in meso and hyperendemic settings (Solomon *et al*. 2003; West *et al*. 2005).

In this population, *C. trachomatis* load increases with disease severity (for both follicular (F) and inflammatory (P) scores), the strongest association being with increasing P-scores. The association between ocular *C. trachomatis* load and disease severity in trachoma is supported by findings from other studies (Burton *et al*. 2003, 2006; Solomon *et al*. 2003; Michel *et al*. 2011). The association between *C. trachomatis* load and high F-scores is in part due to collinearity between F and P, where with high F-scores there is likely to be inflammation present. Inflammation has previously been found to be associated with high *C. trachomatis* loads and persistence of infection in children (Bobo *et al*. 1997). This is consistent with the high prevalence of infection and the spectrum of load and phenotype observed in this age group but may also be associated with pathogen virulence if there are different *C. trachomatis* strains in circulation. Further analysis is underway to investigate associations between pathogen genotype, clinical phenotype and geospatial clustering of strains.

There is spatial dependence in the distribution of *C. trachomatis* infection, demonstrated by positive spatial autocorrelation*.* The distance over which this occurs (1719 m) equates to that of village boundaries. We used this data-driven threshold in our geostatistical models to account for the difficulties in performing this type of analysis in small island populations.

Our data show that the highest burden of infection (and load) is in children under 10 years of age. There are usually only one or two children of this age group within a household in these communities (Last et al. 2014). The high load clusters (HH) and high load outliers (HL) are geographically close to the non-significant clusters and both exist within the village boundaries in this study (<200 m apart). These data support the hypothesis that transmission occurs at village level in this population. Spread from an individual within an HH cluster to an individual in a non-significant cluster is also possible and requires further investigation in longitudinal and mathematical modelling studies.

Although household and village-level clustering is evident in active trachoma and ocular *C. trachomatis* infection (Bailey *et al*. 1989; Blake *et al*. 2009; Hagi *et al*. 2010; Last et al. 2014), the inclusion of this spatial structure in regression models for active trachoma and *C. trachomatis* infection further improves model fit and demonstrates underlying spatial processes in the relationship between infection and disease, such that cases of active trachoma may represent recent exposure to *C. trachomatis* infection. The effect of spatial dependence in infection is greater than in active trachoma, perhaps reflecting the complexity of the disease process, where host–pathogen interactions contribute.

There was no global spatial autocorrelation evident for *C. trachomatis* bacterial load, likely reflecting the stronger influence of underlying social and biological rather than spatial processes. However, we were able to use local indicators of spatial association to examine fine-scale spatial clustering to identify clustering of high load infections. Spatial clusters, independent of age and gender, of individual infections with high bacterial loads (HH) and high load outliers that cluster with other low loads (HL) exist. Furthermore, there was no evidence of clustering of low load infections (LL), suggesting that high load infections may be important in transmission of chlamydial infection. There appears to be a threshold of *C. trachomatis* load below which HH clustering (and being a HL outlier) does not occur. The same phenomena were observed in data including infected and uninfected individuals and data with infected individuals only suggesting that low load infection may represent a negligible risk of transmission. This supports the described Allee effect, which hypothesises that a reduction in chlamydial fitness due to reduced pathogen population size or density results in its disappearance from a population (Chidambaram *et al*. 2005).

There are a limited number of studies that have assessed the spatial distribution of trachoma (Polack *et al*. 2005; Clements *et al*. 2010; Smith et al. 2013) and ocular *C. trachomatis* infection (Broman *et al*. 2006; Yohannan *et al*. 2014). Broman *et al*. (2006) did not find clustering of active trachoma in children under the age of 10 years, but found clusters of households with high bacterial loads (defined as the household mean load) in treatment-naïve communities, supporting the findings in this study. Yohannan *et al*. (2014) examined binary household-level data using a K-function nearest neighbours analysis. The methodology for cluster detection in these studies was heterogeneous, and measures of spatial dependence were not included in these analyses.

This cross-sectional study is limited in the assumption of load and clinical disease severity as a steady state. There are few data addressing stability of *C. trachomatis* load and disease phenotype (Bobo *et al*. 1997). Bobo *et al*. (1997) conducted weekly surveys for 3 months in hyperendemic communities, finding that 62% of children had at least one infection during that time and of those 64% were persistently infected and had higher mean *C. trachomatis* loads and more severe disease than those who were sporadically infected. Additionally, host conjunctival immune response and duration of infection add complexity to the interaction between *C. trachomatis* infection and disease severity (Faal *et al*. 2006; Gambhir *et al*. 2009). *Chlamydia trachomatis* strain diversity may also be associated with bacterial load, the spectrum of disease severity and the clustering of high load infections within these communities.

The role and importance of HH clusters and HL outliers in *C. trachomatis* transmission is speculative, but their presence, and the absence of low load clusters (LL) and low load outliers (LH), suggests that *C. trachomatis* load may have a role in transmission, and that these clusters may represent a source for spread of ocular *C. trachomatis* infection. Studies in trachoma and *C. trachomatis* infection in mouse models have suggested that *C. trachomatis* load is associated with transmission (Pal *et al*. 1998; West *et al*. 2005). There is also some evidence from studies of urogenital *C. trachomatis* infection in humans that individuals with asymptomatic infection have lower *C. trachomatis* loads than individuals with symptomatic infections (Wiggins *et al*. 2009) and that the chance of sexual transmission of *C. trachomatis* per sexual act may be influenced by load (Dirks *et al*. 2015). However, the determinants of *C. trachomatis* load and its role in transmission and the development of sequelae are not well understood (Vodstrcil *et al*. 2014). Further longitudinal data in the context of trachoma endemicity and mass drug treatment are required to fully address these questions and investigate the dynamics of *C. trachomatis* load in transmission.

## CONCLUSION

This is the first study to use individual-level quantifiable *Chlamydia trachomatis* infections from a geospatially representative population-based sample to investigate spatial clustering of *C. trachomatis* infection. These data show that increasing *C. trachomatis* load is related to increasing disease severity in active trachoma, particularly with respect to inflammation. We have provided a global statistical measure for spatial autocorrelation in infection and disease and used local indicators of spatial association to describe the location and nature of the clusters in relation to *C. trachomatis* load.

These data suggest that high load *C. trachomatis* infections cluster spatially and may be important in transmission, although further longitudinal study is required. Further epidemiological and *in vitro* studies are required to provide a more complete picture of the relationship between disease severity and chlamydial load.

These geospatial tools may be useful as tools in trachoma surveillance to identify clusters of infection and thresholds of *C. trachomatis* bacterial load that may be important foci of transmission. Using these methods in conjunction with novel molecular tools to better define *C. trachomatis* strains and ‘virulence’ may also improve our understanding of *C. trachomatis* pathogenesis and transmission.
